# Sleep Quality Among Healthcare Providers During Conflict Crisis in the Gaza Strip: A Cross-Sectional Study

**DOI:** 10.1192/bjo.2026.11202

**Published:** 2026-06-30

**Authors:** Ahmed Alhaj, Mohammed Afana

**Affiliations:** 1 MSF, Gaza, Palestine; 2 MoH, Gaza, Palestine

## Abstract

**Aims:**

Healthcare providers working in conflict zones face unique occupational and psychological challenges that significantly impair sleep quality. In the Gaza Strip, prolonged exposure to violence and humanitarian crises exacerbates these challenges, yet data on the sleep health of this critical workforce remain scarce. This cross-sectional study aimed to assess the prevalence and patterns of sleep disturbances among healthcare providers at Nasser Medical Complex during the 2023–2025 Israel–Gaza conflict, and to examine associations between sleep quality and sociodemographic and occupational factors.

**Methods:**

A cross-sectional study was conducted among 400 healthcare providers (70% nurses, 20% physicians, 10% non-medical staff) at Nasser Medical Complex from May to July 2025. Of 1000 eligible, 993 were approached; 400 participated (participation rate 40.3%). Participants completed the validated Arabic version of the Pittsburgh Sleep Quality Index (PSQI) and a sociodemographic questionnaire. Descriptive statistics, bivariate analyses, and multivariate regression were used to evaluate sleep quality and its predictors. Ethical approval: Palestinian Ministry of Health (Ref 2563158). No funding.

**Results:**

The PSQI demonstrated acceptable internal consistency (Cronbach’s alpha [value pending]). Thirty-five per cent of participants reported poor sleep quality (PSQI >5). Additionally, 40% reported sleeping less than 6–7 hours nightly. Sleep disturbances were frequent, including difficulty initiating sleep (50% reporting problems weekly or more), nighttime awakenings (60%), loud snoring (37.5%), and breathing pauses (20%). Physical discomfort during sleep–such as back pain and breathing difficulties–was prevalent.

**Conclusion:**

Sleep disturbances are alarmingly common among Gaza’s healthcare providers in conflict settings. These findings underscore the urgent need for integrated mental health and sleep interventions, occupational health screenings, and infrastructural support to safeguard provider wellbeing and healthcare delivery sustainability in protracted crises.

